# Identification of the Potential Genes Regulating Seed Germination Speed in Maize

**DOI:** 10.3390/plants11040556

**Published:** 2022-02-19

**Authors:** Huairen Zhang, Jie Zang, Yanqing Huo, Zhaogui Zhang, Huabang Chen, Xunji Chen, Juan Liu

**Affiliations:** 1 State Key Laboratory of Plant Cell and Chromosome Engineering, Innovative Academy of Seed Design, Institute of Genetics and Developmental Biology, Chinese Academy of Sciences, Beijing 100101, China; hrzhang@genetics.ac.cn (H.Z.); zangjiebaiao@163.com (J.Z.); 20200037@wfu.edu.cn (Y.H.); genetics@126.com (Z.Z.); hbchen@genetics.ac.cn (H.C.); 2 Institute of Biotech & Nuclear, Xinjiang Academy of Agricultural Sciences, Urumqi 830091, China

**Keywords:** maize, seed germination, divergent germination speed, time-series transcriptome, GWAS mapping

## Abstract

Seed germination is the crucial stage in plant life cycle. Rapid and uniform germination plays an essential role in plant development and grain yield improvement. However, the molecular mechanism underlying seed germination speed is largely unknown due to the complexity of the dynamic process and the difficulty in phenotyping. Here, we conducted a time-series comparative transcriptome study of two elite maize inbred lines, 72-3 and F9721, with striking difference in seed germination speed, and identified a major locus underlying maize germination speed through genome-wide association analysis (GWAS) of an F_2_ segregation population. Comparative transcriptome study identified 12 h after imbibition (HAI) as the critical stage responsible for the variation in germination speed. The differentially expressed genes (DEGs) between 72-3 and F9721 were mainly enriched in metabolic pathways, biosynthesis of secondary metabolites, oxidoreductase activity pathways, hormone signal transduction, and amino acid transporter activity pathways. GWAS revealed that germination speed was controlled by a major locus on chromosome 1 with the leading SNP as AX-91332814, explaining 10.63% of phenotypic variation. A total of 87 proposed protein-coding genes surrounding the locus were integrated with DEGs. Combined with evidence from the gene expression database and gene synteny with other model species, we finally anchored three genes as the likely candidates regulating germination speed in maize. This study provides clues for the further exploration of genes controlling the maize seed germination speed, thus facilitating breeding of rapid germinated elite lines through marker assistant selection.

## 1. Introduction

Maize (*Zea mays* L.) is a staple crop and primary resource for feed and biofuels. Seed germination is an essential stage in the life cycle of higher plants [1]. The germinating seeds are highly vulnerable to biotic and abiotic factors including pathogen infection, extreme temperatures, drought, light, waterlogging, and salinity [2,3,4,5]. For instance, the frequent low temperature in the northern parts of China often causes collapsed seed germination, resulting in the high cost of planting and great loss in grain yield. Rapid and uniform seed germination is desired to avoid these damages, thus ensuring higher emergence rate and seedling vigor in the field.

Seed germination is a complex process spanning sequential phases from seed recovering from maturation drying to metabolism resuming and cellular preparation for subsequent seedling growth. The dynamic process involves extensive physiological and biochemical events characterized, among others, by carbohydrate metabolism, hormone signal transduction, and redox homeostasis regulation [1,3,6,7,8,9,10,11,12]. As the main storage carbohydrate in seeds, the starch stored in the endosperm serves as the primary sugar source for embryo growth. During germination, the degradation of stored starch is mainly activated by amylase including α-amylase and β-amylase. α-Amylase is de novo synthesized and expressed in the aleurone layer during seed germination in the presence of endogenous gibberellin (GA) from the embryo while β-amylase is present before germination in an inactive form in rice [13]. The α-amylase activity is enhanced to mobilize starch to supply metabolites such as soluble sugar and energy for seed germination under chilling stress [14]. The primary hormones abscisic acid (ABA) and GA are widely known to play essential roles in regulating seed germination [1,11,12,15,16,17]. ABA induces the expression of late embryogenesis abundant proteins and exerts an inhibitory effect on mechanisms triggering precocious and deleterious germination of developing seeds on the mother plant [16]. GA behaves as an activator of germination that counteracts the inhibitory effect of ABA. The spatial-temporal balance of ABA and GA plays a pivotal role in seed germination by favoring dormancy over germination when the ABA/GA ratio is high and the opposite when low [3,18,19]. Reactive oxygen species (ROS) are recognized as crucial mediators of metabolic and molecular events that drive germination [7,20]. Numerous studies have shown that substantial positive relationships exist between ROS activity and seed germination, and spatiotemporal regulation of ROS production acts in concert with hormone signaling to regulate the cellular events involved in cell expansion associated with germination [7,21,22,23,24,25]. Ma et al. [22] demonstrated that a mitochondria-localized small heat shock protein GhHSP24.7 induced the production of ROS by mitochondrial electron transport chain, which activated seed germination in response to temperature. Leymarie et al. [26] revealed that ROS were first localized within the cytoplasm upon the imbibition of non-dormant seeds, then in the nucleus, and finally in the cell wall, suggesting that ROS production was spatio-temporally regulated, and ROS played different roles during seed germination.

High seed vigor is important for agricultural production due to the associated potential for increased growth and productivity. Seed vigor is often controlled by numerous minor genes and affected by multiple factors such as genetic and physical purity, mechanical damage, and physiological conditions [27]. Li et al. (2017) [14] reported that seed priming with salicylic acid and H_2_O_2_ synergistically promoted hormone metabolism and signal transduction, enhancing energy supply and antioxidant enzyme activities under chilling stress, which are closely relevant to chilling injury alleviation and chilling-tolerance improvement in maize seed. Cao et al. (2019) [28] revealed that seed priming with melatonin improved waxy maize seed germination under chilling stress through improving the antioxidant system and starch metabolism, which protected from oxidative damage. Using an artificial aging strategy, Han et al. (2014) [27] identified sixty-five QTLs using single-nucleotide polymorphism markers to map quantitative trait loci (QTLs) in two connected recombinant inbred line maize populations. The candidate genes were involved in the glycolytic pathway, protein metabolism, and signal transduction. Another study based on a high-density genetic map constructed from a 148 BC_4_F_3_ population identified 18 QTLs, and a stable QTL was detected on chromosome 10 with four candidate genes (GRMZM2G074309, GRMZM2G117319, GRMZM2G465812, and GRMZM2G343519), which may be related to seed vigor after artificial aging [29]. Hu et al. (2016) [30] used 243 lines of the intermated B73 × Mo17 recombinant inbred line population for QTL analysis of low-temperature germination ability. Six QTLs controlling low-temperature germination rate were detected on chromosomes 4, 5, 6, 7, and 9, and contribution rate of single QTL explained between 3.39~11.29%. Zhang et al. (2021) [31] performed GWAS on 300 inbred lines genotyped by 43,943 single nucleotide polymorphisms, and a total of 15 significant SNPs were identified to correlate with seed germination under cold stress. Four candidate gene models (Zm00001d010454, Zm00001d010458, Zm00001d010459, and Zm00001d050021) were previously reported to involve plant tolerance to chilling stress and other abiotic stress. All of the findings facilitate the understanding of genetic and molecular mechanisms underlying seed vigor in maize.

Due to the linkage disequilibrium (LD) and the uneven marker distribution in the maize genome, the candidate genes could only be mapped into a roughly large genomic region. Comparative transcriptome analysis provides a global view and an efficient way to further anchor the candidates through the evaluation of gene expression between two contrasting lines. In this study, to decipher the genetic basis underlying seed germination speed in maize, we combined the comparative transcriptomic approach and GWAS to identify the potential causal genes. We divided the germination process into six time points and investigated the time-series transcriptome differences within/between the two lines. Next, we performed GWAS using a 72-3/F9721 derived F_2_ population to identify the underlying genomic locus. Potential candidate genes were annotated and integrated with DEGs from the transcriptome study. This study will guide further exploration of genes responsible for seed germination speed and facilitate marker-assisted selection for breeding rapid germinated lines.

## 2. Materials and Methods

### 2.1. Materials and Germination Assays

To screen materials with different seed germination speed, we initially assessed 1400 inbred maize lines from diverse ecological regions. These inbred lines were planted under the same field conditions in Beijing in the summer of 2016 to remove age and maternal effects in the seeds, and the seeds were harvested at the mature stage with a black layer. For the germination test, 50 uniform seeds from the middle of the ear were first sterilized with 0.1% H_2_O_2_ for 10 min and then rinsed with sterilized water three times. After that, the seeds with the embryo upward were evenly placed in a Petri dish (diameter = 15 cm) on four layers of sterile filter paper moistened with 15 mL sterilized water. The Petri dishes were then incubated in a light incubator with a constant temperature of 28 °C under a 16/8 h (light/dark) photoperiod. An additional 5 mL of sterilized water was added to the Petri dishes every 12 h during the germination process. The seeds were recognized as germinated after radicle emergence of 0.5 mm. Finally, two inbred lines 72-3 and F9721, which showed uniform germination but strikingly different germination speed, were selected as the plant materials. It took 24 h for line 72-3 and 48 h for line F9721 to achieve a 96~100% germination rate.

### 2.2. Measurement of Total Soluble Sugar and Amylase Activity in Germinating Seeds

For soluble sugar content measurement, 1 mL 80% ethanol was added to 0.1 g of seed flour in a 2 mL tube and incubated for 30 min at 75 °C. The above extraction was repeated twice, and all of the supernatant was transferred to a 15 mL centrifuge tube and diluted to 10 mL with ddH_2_O. Colorimetric method of anthrone and sulfuric acid was used to determine the soluble sugar. In brief, 5 mL anthrone and sulfuric acid solution (1 g anthrone in 100 mL sulfuric acid) was added to a 0.5 mL extract in a 10 mL tube and incubated for 2 min at 100 °C. The content of soluble sugar was detected at 630 nm, and quantified as the ratio in 0.1 g flour (%). Amylase extraction and activity measurement was performed following the Amylase Activity Assay Kit (Solarbio; Catalog NO: BC2040). A sample of 0.1 g of tissues was ground into a fine power and then the samples were extracted according to the instruction, and the activity of amylase was detected at 540 nm. Three replicates were set for all measurements.

### 2.3. ABA, GA, and ROS Measurement

For the GA and ABA measurements, 1 g samples were immediately frozen and ground in liquid nitrogen. The ground tissue was extracted with 1 mL of isopropanol/ddH_2_O/hydrochloric acid (V/V/V = 2:1:0.002) extraction solution in a 2 mL tube and incubated for one hour at 4 °C. Then, 1 mL of dichloromethane was added and shaken at 4 °C for one hour. Samples were centrifuged at 13,000 rpm for 20 min at 4 °C, and the supernatant was collected and dried under nitrogen gas. The content of GA or ABA measurements was performed following the instructions in the GA ELISA Kit (Abmart; Catalog No: AB-C11479B) or ABA ELISA Kit (Abmart; Catalog NO: AB-2278B). Three biological replicates were performed for all measurements.

Seed ROS was evaluated as previously described by [32]. To detect hydrogen peroxide (H_2_O_2_), the germinating seeds were cut longitudinally and incubated in the 3, 3′-diaminobenzidine (DAB) solution (1 mg·mL^−1^ DAB in 50 mM Tris-HCl buffer, pH 5.0) in the dark for 16 h. The DAB solutions were replaced with bleaching (70% ethanol) at room temperature after incubation. The DAB staining seeds were shown, and the relative staining intensity was quantified with ImageJ software (https://imagej.nih.gov/ij/index.html; 20 December 2021). Three biological replicates were performed for all measurements.

### 2.4. RNA Sequencing, Differential Gene Expression Analysis, and Enrichment Analysis

To prepare for the transcriptome sequencing, we performed a germination test of the two lines under the same conditions. Fifty uniform seeds from the middle of the ear were placed in a Petri dish (diameter = 15 cm) for the germination test. Three replicates from three different ears were set. For 72-3, germinating seeds at four time points (0, 6, 12, 24 h after imbibition, HAI) were sampled while six time points (0, 6, 12, 24, 36, 48 HAI) of F9721 were sampled for RNA sequencing. Five germinating seeds were sampled for each time point and the whole seeds were used for RNA extraction. Total RNA was extracted using an RNA Easy Fast Kit DP452 (TianGen BioTech Beijing Corporation, Beijing, China). RNA purity, concentration, and integrity were determined using a Nano-Drop TM 2000 spectrophotometer (Thermo Fisher Scientific, Waltham, MA, USA). Libraries of each RNA sample were constructed using Illumina Truseq RNA Sample Prep Kits. The paired-end of 125 bp sequencing was performed with an Illumina HiSeq2500 sequencer. Adapter trimming and low-quality read removing were performed using Fastp software [33]. Reads were aligned to the B73 AGPv4 reference genome (http://www.maizegdb.org/; 7 January 2022) using the Hisat2 aligner [34], removing the 5′ and 3′ low-quality bases. A python module HTSeq was used to quantify the number of reads mapped on a gene with union mode [35]. Raw gene count data were filtered with at least one read and counted across all samples for each gene. Transcripts per million (TPM) values were generated using R software (https://www.r-project.org; 15 December 2021). Differentially expressed gene (DEG) analysis between samples was performed using DESeq2 [36]. DEGs were classified as the adjusted *p*-value ≤ 0.01 and fold change of |log_2_ Ratio| ≥ 2 after correction for multiple testing. Normalized values of all samples were used to compute Pearson correlation coefficients and principal components analysis (PCA). The Kyoto Encyclopedia of Genes and Genomes (KEGG) pathway enrichment analysis was subjected to the KOBAS (http://kobas.cbi.pku.edu.cn; 1 January 2022) to determine the pathways the DEGs are involved in [37]. The AgriGO online website (http://systemsbiology.cau.edu.cn/agriGOv2/; 5 January 2022) was used to perform Gene Ontology analysis. Adjusted *p*-value less than 0.05 was the threshold to determine the statistical significance. The results were visualized using the R ggplot2 package.

### 2.5. SNP Genotyping and GWAS of Genomic Locus for Germination Speed Variation

To identify the genetic locus controlling the speed of seed germination, we generated an F_2_ segregating population with 72-3 as the female parent and F9721 as the male. We performed the germination test using more than 8000 seeds to screen for the fast and slow germinating seeds. In detail, we selected the earliest germinated seeds from the 50 seeds in each Petri dish as the fast germinated individuals. After taking out the fast ones, the remaining seeds were placed back in the incubator, continuing germination. The last germinated seeds from each dish were considered as the slow germinated ones. Finally, we selected total 77 individuals and ten pools as fast, while 78 individuals and five pools were slow. Each pool consisted of ten individuals. A total of 172 samples including the two parental lines were obtained for genotyping. Seed DNA was extracted using an effective genome DNA Extraction Kit (DP350, TianGen BioTech Beijing Corporation, Beijing, China). DNA quality was checked by Nano-Drop 2000 spectrophotometer. Qualified sample DNA was genotyped by Beijing Compass Biotechnology Corporation with an Axiom Maize 56K SNP Array platform. The quality control of SNPs was conducted using PLINK [38]. Markers with minor allele frequency less than 0.01 and missing data greater than 10% were removed, giving a total of 21,303 high-quality SNPs for the population structure analysis and subsequent association mapping.

To exclude the influence of population structure and individual affiliation, PCAs and kinship matrix were calculated using GAPIT3 [39], which were then applied in the linear mixed models. To evaluate the population structure, GLM, CMLM, FarmCPU, and BLINK models were respectively used for the association analysis. The value of fast germinated lines/pools was coded as 1, while the slow germinated lines/pools were 0. The first three principal contents (PCs) were applied as a fixed effect, and an individual kinship matrix was incorporated as a random effect in the CMLM model. The significant association between markers and trait greater than the threshold of −log_10_ (0.05/21,303) = 5.6 was set to reduce the possible false-positive associations. The Manhattan plot and quantile-quantile (Q-Q) plot were visualized by R package ‘CMplot’ [40]. We narrowed the candidate locus into a 2 Mb region with the most significant SNP as the midpoint due to the high LD in the F_2_ population.

## 3. Results

### 3.1. Characterization of Seed Germination Status in the Two Inbred Maize Lines

**Figure 1:**
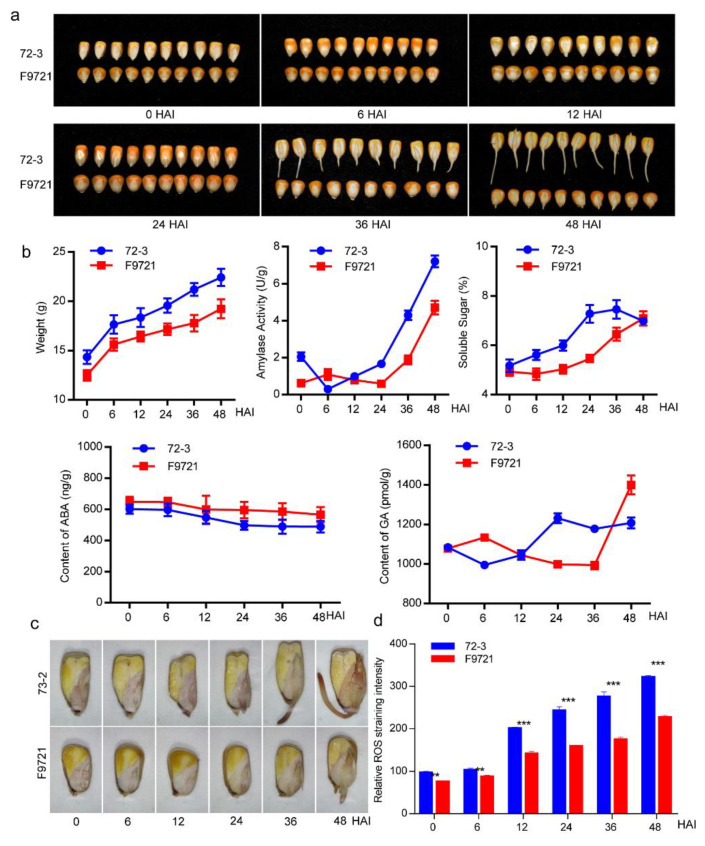
Seed germination process of 72-3 and F9721. (**a**) Comparison of the six germination stages of 72-3 and F9721. (**b**) Dynamic of seed fresh weight, amylase activity, soluble sugar content, and ABA and GA content of the two lines at six germination stages. The content of soluble sugar was quantified as the ratio in 0.1 g flour (%). (**c**) DAB staining for the evaluation of hydrogen peroxide (H_2_O_2_). (**d**) Quantified DAB staining intensity during seed imbibition. Error bars indicate standard deviation (SD), Student’s *t* test; ** *p* < 0.01, *** *p* < 0.001.

We performed preliminary germination tests in 1400 inbred maize lines from diverse ecological regions. The two lines 72-3 and F9721 showed uniform and stable seed germination but with a significantly divergent germination speed of 24 HAI for line 72-3 and 48 HAI for line F9721 (Figure 1a). To investigate the physiological process of seed germination, we measured the fresh seed weight, amylase activity, total soluble sugar content, ROS activity as well as ABA and GA content of the two lines at six consecutive stages (0, 6, 12, 24, 36, and 48 HAI) (Figure 1b). The fresh seed weight of both lines showed continuous increase during imbibition and the fresh weight of 72-3 was consistently higher than that of F9721. In both lines, rapid water uptake occurred within 6 HAI, followed by limited water absorption in the subsequent stages. There was a stable increase in amylase activity in 72-3 after 6 HAI while the increase happened at 24 HAI in F9721, which seemed consistent with the rapid activation of starch degradation in endosperms. The soluble sugar content (%) of 72-3 was higher than that of F9721 after 6 HAI, which is consistent with the dynamic of amylase activity. Meanwhile, we detected the rapid increase in soluble sugar content (%) in 12 HAI of 72-3 and delayed to 24 HAI in F9721. We observed a decrease in ABA content during the germination process of both lines but a significantly lower level of ABA was detected in 72-3 than F9721, confirming the antagonistic role of ABA in seed germination. In contrast, we observed a sharp increase in GA content in the 12 HAI 72-3, which was delayed to 36 HAI in F9721. Taken together, we inferred that 12 HAI was the crucial stage for the activation of metabolism in 72-3, which was delayed to 24 or 36 HAI in F9721. Its worth noting that ROS activity of the two lines demonstrated an obvious increase with the progress of imbibition, and a significantly higher level of ROS activity in 72-3 was detected during the whole germination process (Figure 1c,d). The results may reveal a tight correlation of redox homeostasis with the divergent germination speed between 72-3 and F9721.

### 3.2. Transcriptome Dynamics during the Germination Process of 72-3 and F9721

To study the overall transcriptome dynamics during seed germination, we undertook a time-series transcriptome study of the germinating seeds in 72-3 (0, 6, 12, 24 HAI) and F9721 (0, 6, 12, 24, 36, 48 HAI). Clean reads were well mapped to the B73 APGv4 reference genome (Supplementary Table S1). PCA of the 30 samples and Pearson correlation analysis showed dynamic divergence between different groups and high correlation within each group (Supplementary Figure S1a,b). We then analyzed the DEGs between the imbibing seeds (6, 12, 24, 36, 48 HAI) and the dry seeds (0 HAI) during the germination process. There were respectively 321 (6 vs. 0 HAI), 1862 (12 vs. 0 HAI) and 6969 (24 vs. 0 HAI) DEGs for 72-3 while there were 321 (6 vs. 0 HAI), 809 (12 vs. 0 HAI), 2301 (24 vs. 0 HAI), 4394 (36 vs. 0 HAI), and 5023 (48 vs. 0 HAI) DEGs for F9721 (Supplementary Figure S2a–h). The results showed a consistent increase in DEGs with the progress of germination, and the relatively less DEGs (321 genes) between 6 HAI and 0 HAI in both lines suggested a similar biological status between early imbibing seeds and dry seeds. Additionally, the number of DEGs in 12 HAI 72-3 was much higher than that of F9721, but close to 24 HAI F9721, indicating that 12 HAI might be the critical stage underlying the germination speed divergence between the two lines.

**Figure 2:**
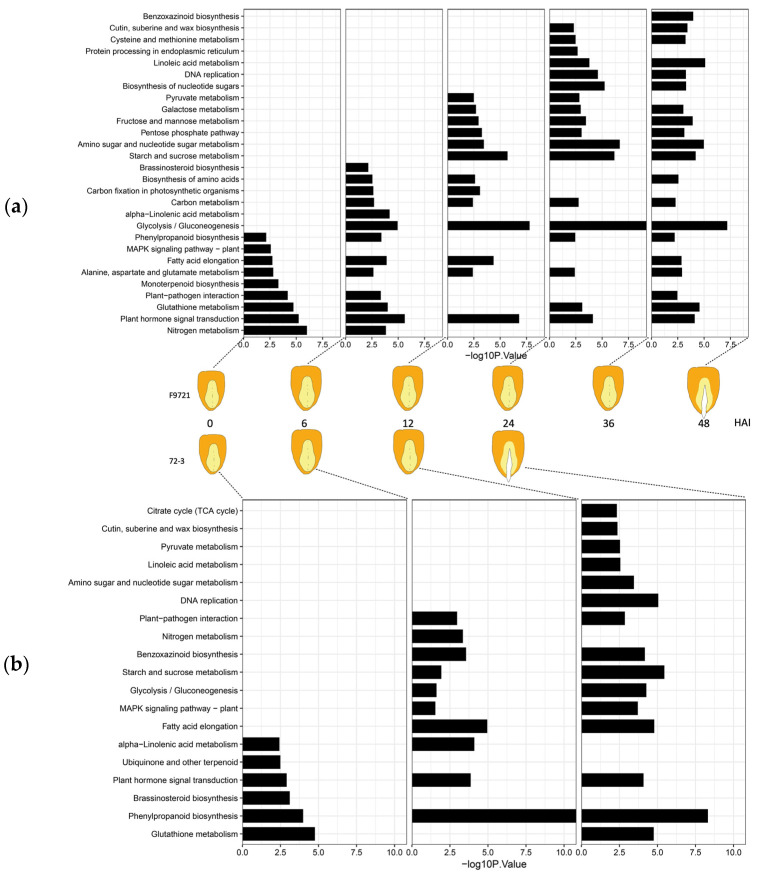
Time series (0, 6, 12, 24 HAI of line 72-3 and 0, 6, 12, 24, 36, 48 HAI of line F9721) transcriptome dynamics indicated by KEGG of DEGs between imbibing and dry seeds. (**a**) Time series (0, 6, 12, 24, 36, 48 HAI) transcriptome dynamics of F9721 between imbibing and dry seeds. (**b**) Time series (0, 6, 12, 24 HAI) transcriptome dynamics of 72-3 between imbibing and dry seeds.

To gain further insight into the biological pathways these DEGs are involved in, we performed KEGG analysis at each time point (Figure 2a,b). For 6 HAI at early imbibition, DEGs were mainly enriched in the processes of glutathione metabolism, hormone signal transduction, and phenylpropanoid biosynthesis in both lines, which may reflect the break of dormancy and initiation of imbibition. We also detected two additional pathways of brassinosteroid biosynthesis and alpha-linolenic acid metabolism enriched in 6 HAI of 72-3, but were delayed to 12 HAI in F9721, which may imply the correlation of these pathways with the germination divergence of the two lines. At 12 HAI, the two lines showed enrichment in processes of hormone signal transduction, phenylpropanoid biosynthesis, plant–pathogen interaction, fatty acid elongation, and alpha-linolenic acid metabolism, which revealed the initiation of germination in both lines. Notably, starch and sucrose metabolism, the typical germination events providing the primary sugar source for embryo growth, was highly enriched in 12 HAI of 72-3 but delayed to 24 HAI of F9721. This further strengthened our judgement that 12 HAI was the major stage controlling the divergent germination speed of 72-3 and F9721. At 24 HAI when 72-3 germinated but F9721 was still under imbibition, DEGs of both lines were enriched in the processes including hormone signal transduction, starch and sucrose metabolism, fatty acid elongation, amino sugar and nucleotide sugar metabolism, and pyruvate, which reflected an overall activation of metabolic pathways and physiological processes required for germination. Consistent with the fast germination of 72-3, we also detected that four pathways occurred in 24 HAI of 72-3, but delayed to 36 HAI in F9721 (i.e., DNA replication, linolenic acid metabolism, cutin, suberine, and wax biosynthesis as well as phenylpropanoid biosynthesis), which were required for the growth of the embryo and the rapid morphogenesis of seedlings. For 36 and 48 HAI of F9721, DEGs were enriched in similar pathways such as those in 24 HAI of 72-3, from which we inferred that germination of F9721 was actually started-up in 36 HAI seeds, even if we could not observe the protrusion of the radicle.

### 3.3. Time-Series Analysis of DEGs between 72-3 and F9721

**Figure 3:**
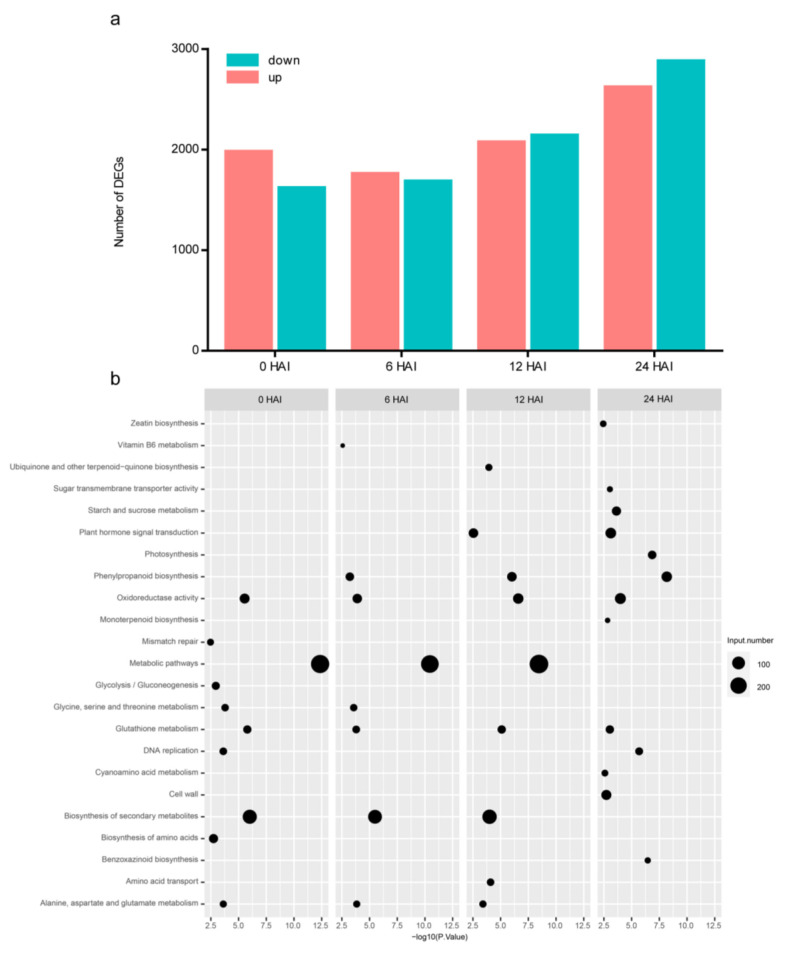
Transcriptome comparisons between 72-3 and F9721 during seed germination. (**a**) The number of DEGs between 72-3 and F9721 at the indicated stages. (**b**) KEGG analysis of DEGs between 72-3 and F9721 at indicated stages.

To investigate the genetic basis underlying the divergence of germination speed between 72-3 and F9721, we conducted a time-series transcriptome comparison between the two lines at 0, 6, 12, and 24 HAI. As expected, a large number of DEGs were detected across the four time points and the DEG number (72-3 vs. F9721) was markedly increased at 12 HAI and 24 HAI (i.e., 4174 genes (2121 up and 2053 down) at 12 HAI and 5460 genes (2860 up and 2600 down) at 24 HAI) (Figure 3a and Supplementary Figure S3a–d). KEGG analysis were then performed to investigate the pathways those DEGs participated in (Figure 3b). At 0 HAI and 6 HAI, the DEGs was equally distributed and enriched in three main pathways: metabolic pathways, biosynthesis of secondary metabolites, and oxidoreductase activity pathways. At 12 HAI, besides the three main pathways, hormone signal transduction, photosynthesis, and amino acid transporter activity pathways were also enriched. Interestingly, the markedly enriched metabolic and biosynthesis of secondary metabolite pathways at 0, 6, and 12 HAI were not detected at 24 HAI, and this could be explained by the fact that the metabolic pathways required for germination were activated in F9721 at 24 HAI. These results further proved that 12 HAI and 24 HAI were respectively the crucial stages for the start-up of germination for 72-3 and F9721, and 12 HAI is likely to be the major stage for the germination speed divergence of the two lines. Additionally, the oxidoreductase activity pathway was markedly detected across the four time points. Combined with the fact that ROS activity was substantially higher in 72-3 than F9721 during the whole germination process, we concluded that ROS homeostasis plays an essential role in the divergence of germination speed between 72-3 and F9721.

### 3.4. GWAS of Candidate Locus Responsible for Seed Germination Variation

**Figure 4:**
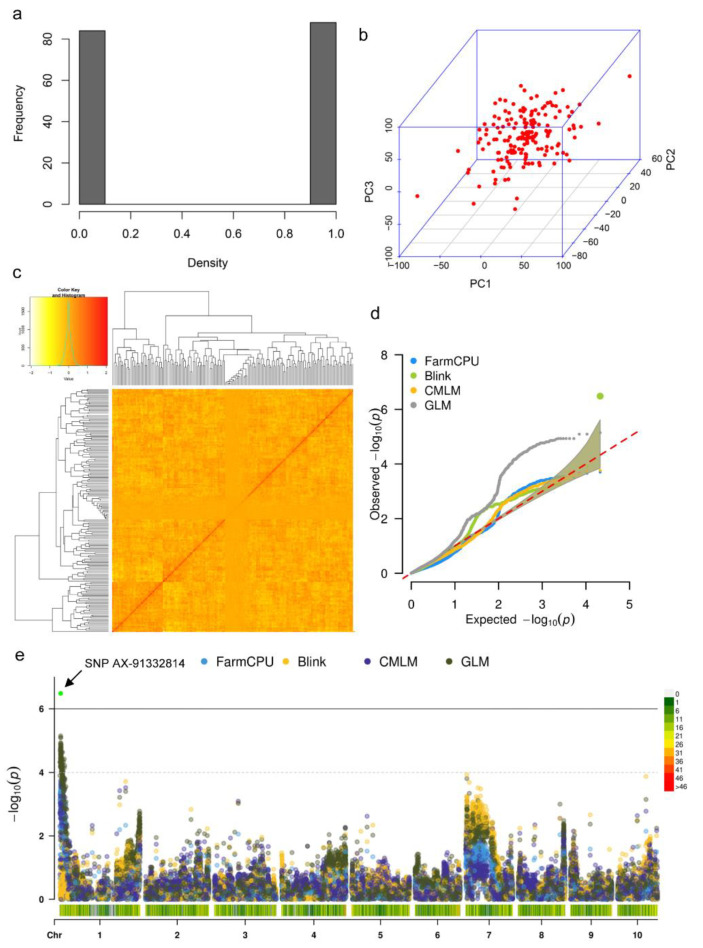
GWAS results of the seed germination speed in the F_2_ population derived from line 72-3 and line F9721. (**a**) The distribution histogram of selected F_2_ lines, 1 indicates the fast germinated individuals/pools, and 0 indicates the slow germinated individuals/pools. (**b**) 3-Dimensional scatter plot of first three PCA. (**c**) Pair-wise individual relatedness matrix computed by GAPIT3.0. (**d**) The quantile-quantile (Q-Q) plot of multiple GWAS models for seed germination speed. (**e**) Manhattan plot of association using four GWAS models, the bottom heatmap shows the SNP density on the ten chromosomes of maize genome.

To elucidate the genetic locus underlying the germination speed divergence, we developed an F_2_ segregating population using line 72-3 as the female parent and line F9721 as the male. The individuals showed dynamic germination speed, and the ones with extremely fast and slow germination speed were strictly selected as the candidates for DNA extraction and SNP genotyping (Figure 4a). In total, 172 samples including the two parental lines were genotyped with an Axiom Maize 56K SNP Array platform. There was a total of 21,303 SNP loci used for the association analysis after the filtration of those loci with minor allele frequency and missing rate. We tested different association models from the GAPIT3 package to obtain the significant marker-trait associations. GLM and CMLM models are two single-locus models, and CMLM accounts for both population structure and relatedness while GLM considered only the population structure. Additionally, we employed two multi-locus models, FarmCPU and BLINK, to improve the statistic power. PCA of population structure showed no prominent cluster of individuals while the kinship matrix showed two main clusters in the population (Figure 4b,c).

**Figure 5:**
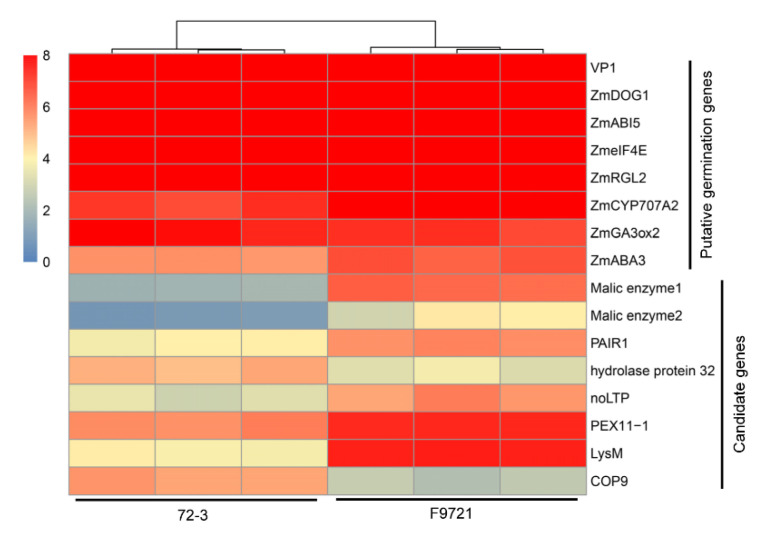
Gene expression of putative germination genes and GWAS candidate genes. Putative germination genes indicate those involved in maize seed germination or maize homologs with germination genes of *Arabidopsis*. Candidate genes indicated DEGs located in the 2 Mb GWAS candidate locus.

Q-Q plot showed that CMLM, FarmCPU, and BLINK could significantly reduce the false positives in the association test (Figure 4d). Using the standard of −log_10_ (0.05/21303) ≥5.6, both single-locus and multi-locus analyses mapped one significant locus with the leading SNP AX-91332814 located on chromosome 1, explaining 10.63% of phenotypic variation (Figure 4e). Due to the high LD decay in the F_2_ population, we finally designated the 2 Mb region surrounding AX-91332814 (1 Mb upstream and 1 Mb downstream) as the candidate locus. In total, 87 protein-coding genes were proposed according to the B73 APGv4 reference genome. To determine the causal genes, we integrated the 87 genes with DEGs of 12 HAI and found seven genes showing significantly differential expression (Figure 5). After evaluation of the gene expression pattern and potential function synteny with model plants including rice and *Arabidopsis*, we finally mapped three candidate genes for maize germination speed variation and their putative biological function was listed (Supplementary Table S2).

## 4. Discussion

Seed germination is one of the most crucial stages in the plant life cycle. Uniform and rapid germination plays a critical role in subsequent plant development and yield improvement. Thus, massive agricultural techniques such as priming and seed coating have been developed to improve seed germination performance in maize cultivation [14,28,41]. Seed germination is completed by radicle emergence, which is determined by the balance of the growth potential of the embryo and the mechanical resistance of the covering tissues such as the endosperm and the testa [8]. An active embryo cannot emerge until the barrier of the covering tissues are overcome, which coordinately control the seed germination speed. For different inbred lines, maize demonstrated great variations in seed germination speed, however, the underlying genetic basis have not yet been identified. This may be attributed to the complexity of the rapid transition process from dry seeds to seedlings within a short time period and the difficulty in the phenotyping of field germinating seeds. Using a water culture method, we preliminarily screened 1400 diverse inbred lines from different ecological regions and found that they demonstrated great variations in both germination rate and germination speed, ranging from 20 h to 80 h to achieve a ≥90% germination rate. We finally selected two elite inbred lines 72-3 and F9721 as the plant materials due to their uniform and high germination rate (96–100%), but with markedly divergent germination speed of 24 h for 72-3 and 48 h for F9721.

Seed germination begins with rapid water imbibition by the quiescent dry seed, followed by a period with limited water uptake and terminates with the protrusion of radicle from the seed coat [8]. During seed desiccation, amino acids and sugars accumulate in mature, dry seeds, which will be used for seed germination. After seed imbibition, amino acids and related organic acid contents were increased continuously. The phosphorylated sugars, fructose-6-phosphate and glucose-6-phosphate, were extremely increased, indicating activation of catabolism [42]. In our study, we detected a significant increase in soluble sugar content during seed germination of both 72-3 and F9721, indicating the rapid activation of starch degradation in imbibing seeds. ROS such as hydrogen peroxide (H_2_O_2_), superoxide ion (O_2_•), and hydroxyl radicals (OH•) are normally produced during cell metabolism. ROS plays a dual role in plant physiological and developmental processes and resist stress, which depends on its concentration, physiological conditions, and the specificity of the process [43,44,45]. Verma et al. (2015) [46] postulated that H_2_O_2_ and ROS production during germination contributed to reserve mobilization through oxidative modifications of stored proteins, which may be recognized by storage organs as signals to mobilize reserves to the rapidly growing axis. Diaz-Vivancos et al. (2013) [47] concluded that ROS plays a vital role in seed proteome and transcriptome remodeling by selective oxidation, triggering dormancy release and germination. We showed that ROS activity of both 72-3 and F9721 was markedly increased with the progress of imbibition, and ROS activity was significantly higher in 72-3 than that in F9721, indicating a close relationship of redox homeostasis with germination speed discrepancy between 72-3 and F9721.

Previous studies have compared the transcriptome profiling between two maize inbred lines at two stages, revealing a markedly transcriptome changes between different lines as well as different stages [48]. In this study, we investigated the germination process of 72-3 and F9721 at six time points, combined with comparative transcriptome analysis, and recognized 12 HAI as the crucial stage for germination speed divergence between the two lines. The illustration of key stages for seed germination speed divergence will facilitate exploring potential genes responsible for germination speed variation. It was well documented that GA and ABA act antagonistically in regulating seed germination and that ABA and GA levels change substantially during seed imbibition. The biosynthesis of the two phytohormones are highly coordinated with the accumulation of GA and a reduction in ABA levels during seed germination [1,10,11,12,15,16,17]. We then checked the expression pattern of maize homologues to those *Arabidopsis* dormancy/germination-associated genes involved in the ABA and GA metabolism and signaling pathway (Figure 5). Among them, ABI3 and CYP707A2 contribute to ABA biosynthesis and thereby regulate seed dormancy release and subsequent germination [19]. ABI5 encodes a transcription factor playing crucial roles in seed germination by directly binding to the ABA-responsive element to regulate their expression and inhibit seed germination [49,50,51]. GIBBERELLIN 3-OXIDASE (GA3ox2) promotes GA biosynthesis and the DELLA protein REPRESSOR OF ga1-3-LIKE2 (RGL2) plays an important role in repressing seed germination by regulating GA signaling and mediating the interaction of GA and ABA during seed germination [52,53,54,55]. The initiation factor eIF4E was reported to regulate the translation of mRNAs during seed germination [56]. Maize Viviparous1 (VP1) is the ortholog of Arabidopsis ABI3 transcription factor that regulates seed development and germination through ABA signaling [57,58]. DELAY OF GERMINATION1 (DOG1) is a key gene required to induce and maintain seed dormancy by promoting ABI5 expression and depressing the activity of the PP2C phosphatases AHG1 and AHG3 [8,59,60,61,62,63,64]. All these genes were highly expressed in 72-3 and F9721 without significant difference, excluding their crucial roles in the determination of germination speed variation between the two lines. For the seven DEGs located in the GWAS locus, we also comprehensively investigated their expression pattern and putative biological function, and finally mapped the three most likely candidates with significantly differential expression between 72-3 and F9721. Zm00001d011289 and Zm00001d012623 both encode putative malic enzymes, catalyzing the oxidative decarboxylation of malate to pyruvate and are required to protect seeds against oxidation during seed dry storage [65]. Zm00001d048502 is a putative CONSTITUTIVE PHOTOMORPHOGENIC (COP) gene acting as a central regulator of seed dormancy and germination that functions in the light signaling pathway in Arabidopsis [66]. However, the function of the three genes needs further validation and the other four DEGs cannot be ruled out.

Seeds accumulate mRNAs during seed maturation, which are known as ‘stored mRNAs’ or ‘long-lived mRNAs’ due to their ability to remain translatable over extended periods of time even under stress conditions [67,68]. On imbibition, seeds transform from a quiescent dry state to a fully active metabolic state, and selectively translate subsets of these stored mRNAs during seed germination [69,70,71]. Stored mRNAs are found in most angiosperms and are believed to be crucial for protein synthesis during seed germination. Microarray analysis has detected more than 12,000 and 17,000 different types of stored mRNAs present in the mature dry seeds of Arabidopsis and rice, which account for 45% and 42.5% of genes transcribed by the Arabidopsis and rice genome, respectively [68]. Our transcriptome study identified 25,292 and 26,131 stored mRNAs in line 72-3 and line F9721, respectively, which account for 66.6% of genes transcribed by the maize genome and is much higher than those of Arabidopsis and rice. Further analysis showed that a total of 3558 stored mRNAs were differentially expressed between 72-3 and F9721. GO enrichment analysis showed that these DEGs are mainly involved in cell surface receptor linked signaling pathways, intracellular signaling pathways, peptidyl-amino acid modifications, and DNA recombination (Supplementary Figure S4a). Previous studies showed that stored mRNAs are not only important for early protein synthesis, but are also translated at later stages of germination, showing that they play a role in both early and late stages of seed germination [72]. Our study also revealed that both the upregulated and the downregulated genes showed a consistent expression pattern during the whole process of germination, implying that they might play a role in the discrepancy of germination speed between 72-3 and F9721 (Supplementary Figure S4b,c). The results provide clues for the further mechanical study of stored mRNAs during maize seed germination.

In conclusion, although seed dormancy and germination has long been concerned and well-studied, the knowledge has mainly centered on the model plant *Arabidopsis.* With the frequent occurrence of abnormal weather conditions such as drought, flood, and especially the late spring coldness, good germination and seedling performance has become one of the hotspots of concern by farmers and breeders to decrease the planting costs and loss of grain yields of crop. Our study provided a primary global view of transcriptome changes during seed germination. Through the physiological and transcriptome comparison of fast germinated line 72-3 and slow germinated line F9721, we deciphered the crucial role of both the hormone regulatory pathway and ROS homeostasis regulatory pathway in the divergence of seed germination speed between 72-3 and F9721. Combined with GWAS, we mapped a new locus on 2,941,215 bp of chromosome 1 with the leading SNP as AX-91332814. Integration and functional prediction of DEGs located in the GWAS locus finally identified three potential causal genes regulating the germination speed variation in maize. This study provides a basis for the further identification of genes controlling maize seed germination speed, and facilitates the breeding of rapid germinating lines through marker assistant selection.

## Data Availability

The raw RNA sequencing data and expression TPM matrix were deposited in the National Center for Biotechnology Information Gene Expression Omnibus (http://www.ncbi.nlm.nih.gov/projects/geo/; 10 January 2022) under accession number GSE193292.

## Supplementary Materials

The following supporting information can be downloaded at: https://www.mdpi.com/article/10.3390/plants11040556/s1. Table S1: Summary of reads alignment with the B73 reference genome APGv4. Table S2: List of proposed genes within the 2 Mb candidate locus. Figure S1: Overall analysis of the transcriptome data. Figure S2: Volcano plots of DEGs between different imbibition stages. Figure S3: Volcano plots of DEGs between 72-3 and F9721. Figure S4: Expression pattern of differentially expressed stored mRNAs and GO enrichment.

## Abbreviations

SNP	Single nucleotide polymorphism
QTL	Quantitative traits loci
ROS	Reactive oxygen species
PCA	Principal components analysis
FDR	False discovery rate
GLM	General linear model
CMLM	Compressed mixed linear model
FarmCPU	Fixed and random model circulating probability unification
BLINK	Bayesian-information and linkage-disequilibrium iteratively nested keyway
GWAS	Genome-wide association analysis
GAPIT	Genomic association and prediction integrated tool
LD	Linkage disequilibrium
