# Supplementary material for: Identification of the Potential Genes Regulating Seed Germination Speed in Maize
Source: Plants (Basel). 2022 Feb 19;11(4):556. doi: 10.3390/plants11040556 (PMC8879924; doi:10.3390/plants11040556)
Supplement: Supplementary file 1 [file plants-11-00556-s001.zip › Supplementary_Figure_Table/Supplementary_Figure_Table.pdf]

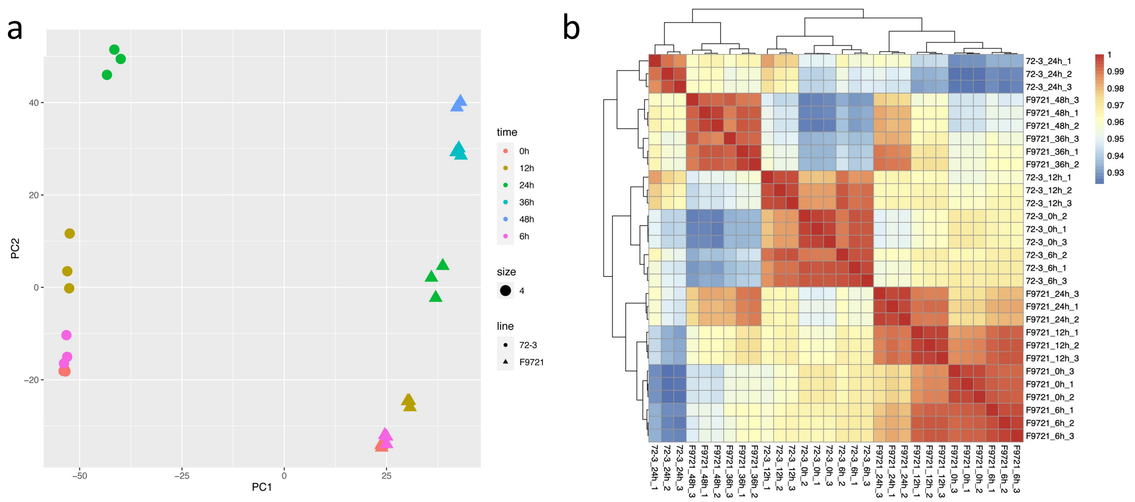

Supplementary Figure S1: Overall analysis of the transcriptome data. (a) PCA plot of the two inbred lines transcriptome change during seed germination. (b) Pearson correlation coefficients heat map between samples from 72-3 and F9721 at six germination stages.

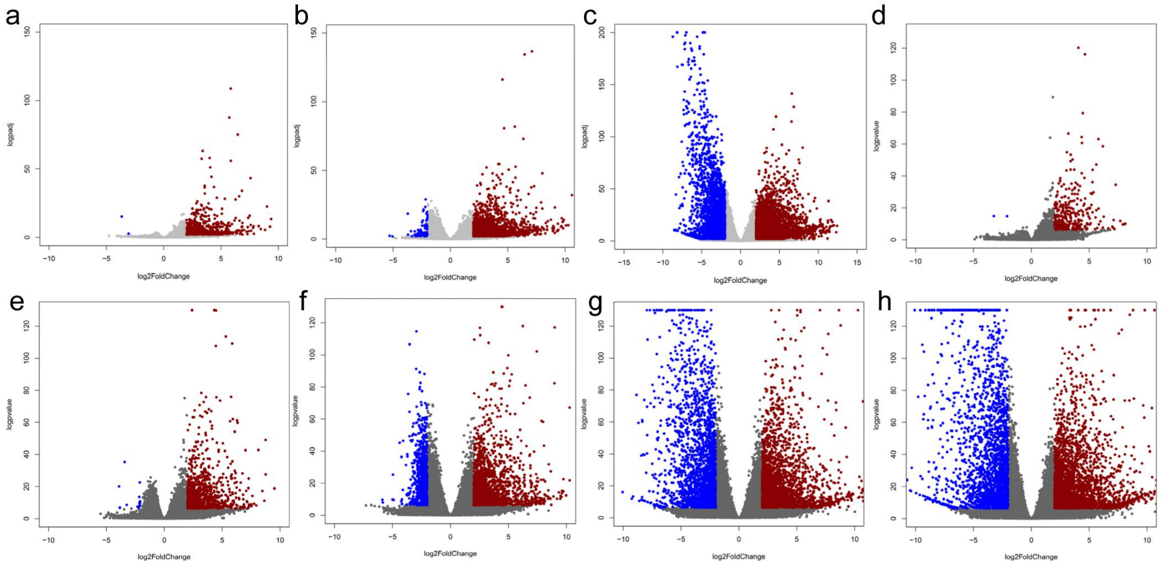

Supplementary Figure S2: Volcano plots of DEGs between different imbibition stages. (a) Volcano plot of DEGs for 72-3 6 HAI versus 0 HAI. (b) Volcano plot of DEGs for 72-3 12HAI versus 0 HAI. (c) Volcano plot of DEGs for 72-3 24 HAI versus 0 HAI. (d) Volcano plot of DEGs for F9721 6 HAI versus 0 HAI. (e) Volcano plot of DEGs for F9721 12 HAI versus 0 HAI. (f) Volcano plot of DEGs for F9721 24 HAI versus 0 HAI. (g) Volcano plot of DEGs for F9721 36 HAI versus 0 HAI. (h) Volcano plot of DEGs for F9721 48 HAI versus 0 HAI.

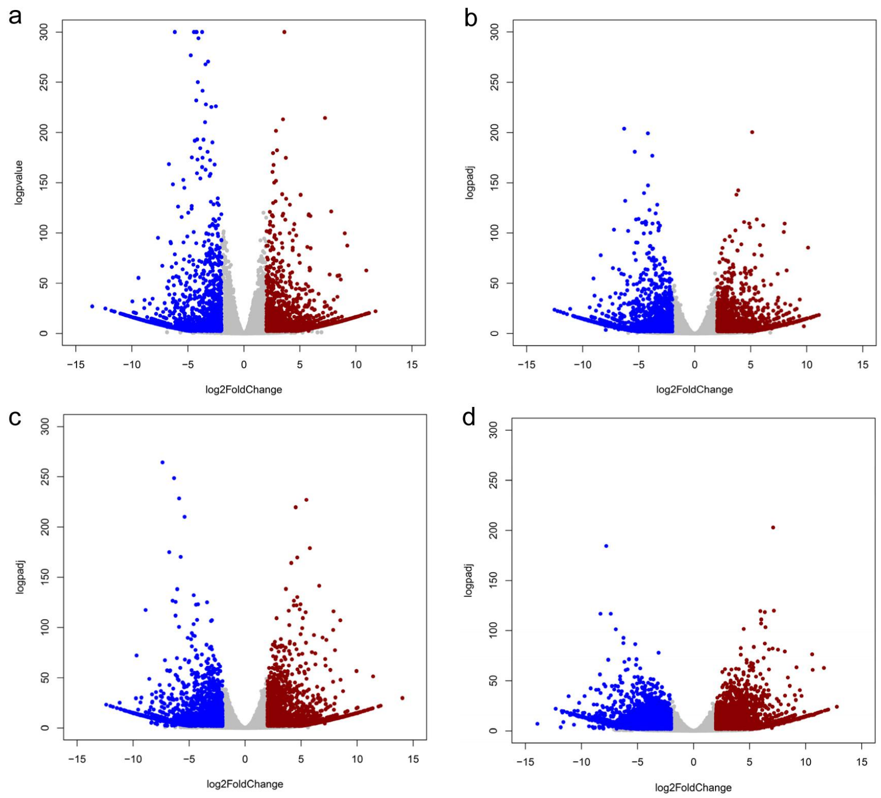

Supplementary Figure S3: Volcano plots of DEGs between 72-3 and F9721 (a) Volcano plot of DEGs for 72-3 versus F9721 at 0 HAI. (b) Volcano plot of DEGs for 72-3 versus F9721 at 6 HAI. (c) Volcano plot of DEGs for 72-3 versus F9721 at 12 HAI. (d) Volcano plot of DEGs for 72-3 versus F9721 at 24 HAI.

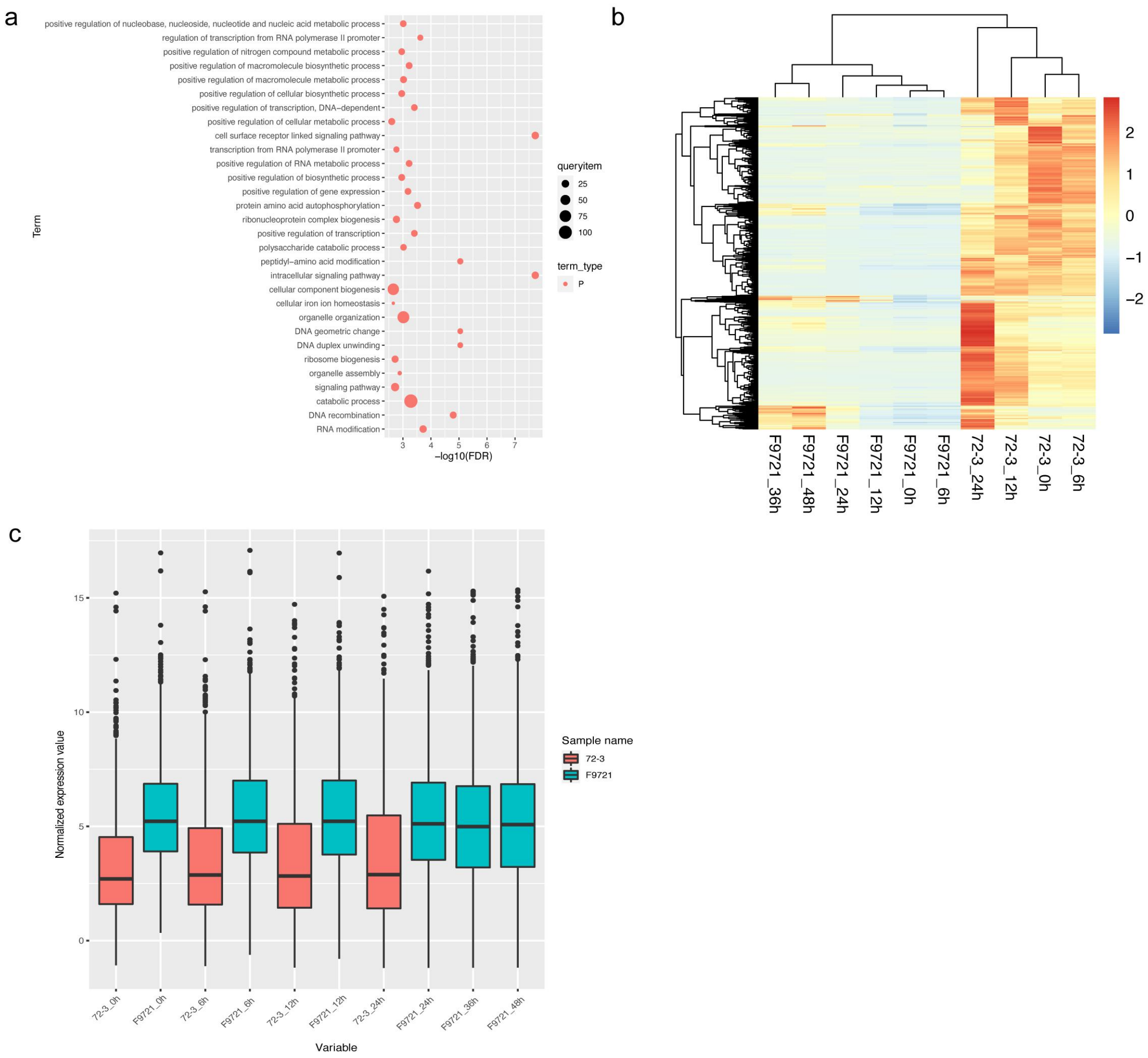

Supplementary Figure S4: Expression pattern of differential expressed stored mRNAs and GO enrichment. (a) GO enrichment of differential expressed stored mRNAs in dry seeds between 72-3 and F9721. (b) Expression pattern of up-regulated stored mRNAs in 72-3 versus F9721 during seed germination. (c) Expression pattern of down-regulated stored mRNAs in 72-3 versus F9721 during seed germination.

Supplementary Table S1: Summary of reads alignment with B73 reference genome APGv4

Samples	Pair-end reads number	GC content (%)	Mapping rate
72-3_0h_1	11655657;11655657	54;54	92.78%
72-3_0h_2	11791262;11791262	54;54	92.66%
72-3_0h_3	12061984;12061984	53;53	92.78%
72-3_12h_1	17835078;17835078	53;53	93.25%
72-3_12h_2	14910260;14910260	55;55	93.92%
72-3_12h_3	11815028;11815028	54;54	92.97%
72-3_24h_1	16773188;16773188	54;54	95.12%
72-3_24h_2	12216469;12216469	53;52	94.22%
72-3_24h_3	14059372;14059372	53;53	95.62%
72-3_6h_1	13305083;13305083	55;54	93.00%
72-3_6h_2	14112810;14112810	56;56	93.54%
72-3_6h_3	13893967;13893967	55;55	93.23%
F9721_0h_1	15375316;15375316	55;55	90.06%
F9721_0h_2	12888583;12888583	55;55	88.53%
F9721_0h_3	14573350;14573350	54;54	88.31%
F9721_12h_1	17303918;17303918	57;57	88.99%
F9721_12h_2	17143408;17143408	56;56	89.57%
F9721_12h_3	14655576;14655576	55;55	88.58%
F9721_24h_1	13682849;13682849	55;55	90.04%
F9721_24h_2	14605629;14605629	55;55	88.17%
F9721_24h_3	15558163;15558163	55;55	89.49%
F9721_36h_1	13495568;13495568	53;53	90.44%
F9721_36h_2	14579617;14579617	54;54	89.99%
F9721_36h_3	13066391;13066391	51;51	91.06%
F9721_48h_1	12172336;12172336	54;53	91.20%
F9721_48h_2	13249328;13249328	54;54	90.70%
F9721_48h_3	13868005;13868005	51;51	90.92%
F9721_6h_1	12888169;12888169	54;54	88.95%
F9721_6h_2	15938142;15938142	56;56	88.63%
F9721_6h_3	15904529;15904529	56;56	88.96%

Supplementary Table S2: Gene list of 2 Mb candidate locus

Gene id	Description
Zm00001d004505	drug resistance transporter-like ABC domain protein
Zm00001d007916	uncharacterized
Zm00001d011289	Malic enzyme1
Zm00001d012623	Malic enzyme 2
Zm00001d018978	uncharacterized
Zm00001d025415	phosphoprotein phosphatase inhibitors
Zm00001d027292	scarecrow-like protein 6
Zm00001d027293	signal recognition particle receptor homolog 1
Zm00001d027295	beta tubulin1
Zm00001d027296	DUF3527 domain protein
Zm00001d027298	uncharacterized LOC100275685
Zm00001d027299	mediator of RNA polymerase II transcription subunit 12-like
Zm00001d027300	protein PAIR1
Zm00001d027302	uncharacterized
Zm00001d027303	Pleiotropic drug resistance protein 2
Zm00001d027304	ATPase 9, plasma membrane-type
Zm00001d027305	uncharacterized
Zm00001d027306	F-box protein
Zm00001d027307	cingulin
Zm00001d027308	RING/U-box superfamily protein
Zm00001d027309	uncharacterized
Zm00001d027310	vegetative cell wall protein gp1
Zm00001d027311	uncharacterized
Zm00001d027312	putative RING zinc finger domain superfamily protein
Zm00001d027313	hydrolase protein 32
Zm00001d027314	uncharacterized
Zm00001d027315	uncharacterized
Zm00001d027317	rolled leaf 2
Zm00001d027318	uncharacterized
Zm00001d027319	4-phosphopantetheinyl transferase sfp
Zm00001d027320	CemA-like proton extrusion protein-related
Zm00001d027321	putative peptidyl-prolyl cis-trans isomerase family protein
Zm00001d027322	Protein weak chloroplast movement under blue light 1
Zm00001d027323	uncharacterized
Zm00001d027324	THO complex subunit 1
Zm00001d027325	peptidoglycan-binding LysM domain-containing protein
Zm00001d027326	uncharacterized
Zm00001d027329	DNA cytosine methyltransferase Zmet 3
Zm00001d027330	uncharacterized
Zm00001d027332	Nonspecific lipid-transfer protein

Zm00001d027333	uncharacterized
Zm00001d027334	uncharacterized
Zm00001d027335	trihelix transcription factor GTL1
Zm00001d027337	kinesin-like protein KIN-14E
Zm00001d027338	uncharacterized
Zm00001d027339	RNA binding protein
Zm00001d027340	ATP binding protein
Zm00001d027341	ATP binding protein
Zm00001d027342	uncharacterized
Zm00001d027343	fertility restorer
Zm00001d027344	molybdate-anion transporter
Zm00001d027345	mitochondrial import inner membrane translocase subunit TIM23-2
Zm00001d027346	early nodulin-like protein 1
Zm00001d027347	uncharacterized
Zm00001d027348	uncharacterized
Zm00001d027349	pentatricopeptide repeat-containing protein At3g53170
Zm00001d027350	uncharacterized
Zm00001d027351	uncharacterized
Zm00001d027352	Pyridoxine/pyridoxamine 5'-phosphate oxidase 2
Zm00001d027353	retinol dehydrogenase 12
Zm00001d027354	mitochondrial inner membrane protein OXA1-like
Zm00001d027355	membrane protein
Zm00001d027359	uncharacterized
Zm00001d027360	Probable protein phosphatase 2C 33
Zm00001d027361	ATPP2-A13
Zm00001d027362	uncharacterized
Zm00001d027363	uncharacterized
Zm00001d027365	vacuolar protein 8
Zm00001d027366	Peroxisomal membrane protein PEX11-1
Zm00001d027367	riboflavin biosynthesis protein ribD
Zm00001d027368	xyloglucan endotransglucosylase/hydrolase protein 5
Zm00001d027369	Protein-ribulosamine 3-kinase chloroplastic
Zm00001d027370	uncharacterized LOC100192905
Zm00001d027371	putative TCP-1/cpn60 chaperonin family protein
Zm00001d039633	uncharacterized
Zm00001d040069	Probable glucuronoxylan glucuronosyltransferase IRX7
Zm00001d047177	TATA-binding protein-associated factor BTAF1
Zm00001d048502	COP9 signalosome complex subunit 1
Zm00001d048507	uncharacterized
Zm00001d048510	uncharacterized
Zm00001d048511	AP-2 complex subunit alpha-2
Zm00001d048527	rolled leaf 1
Zm00001d048536	SKP1-like protein 21

Zm00001d048541	uncharacterized
Zm00001d049814	uncharacterized
Zm00001d051566	LOC100191766-like pseudogene
Zm00001d051742	uncharacterized
